# Role of sirtuin-1 in diabetic nephropathy

**DOI:** 10.1007/s00109-019-01743-7

**Published:** 2019-02-01

**Authors:** Wanning Wang, Weixia Sun, Yanli Cheng, Zhonggao Xu, Lu Cai

**Affiliations:** 1 Department of Nephrology, The First Hospital of Jilin University, 71 Xinmin Street, Changchun, 130021 Jilin Province China; 2 Pediatric Research Institute, Department of Pediatrics, The University of Louisville School of Medicine, Louisville, KY 40292 USA; 3 Departments of Radiation Oncology, Pharmacology and Toxicology, The University of Louisville School of Medicine, 570 S. Preston Str., Baxter I, Suite 304F, Louisville, KY 40292 USA

**Keywords:** Sirtuin-1, Deacetylase, Diabetic nephropathy, Signaling pathway, Pathogenesis

## Abstract

Diabetic nephropathy (DN) is a research priority for scientists around the world because of its high prevalence and poor prognosis. Although several mechanisms have been shown to be involved in its pathogenesis and many useful drugs have been developed, the management of DN remains challenging. Increasing amounts of evidence show that silent information regulator 2 homolog 1 (sirtuin-1), a nicotinamide adenine dinucleotide (NAD+)–dependent protein deacetylase, plays a crucial role in the pathogenesis and development of DN. Clinical data show that gene polymorphisms of sirtuin-1 affect patient vulnerability to DN. In addition, upregulation of sirtuin-1 attenuates DN in various experimental models of diabetes and in renal cells, including podocytes, mesangial cells, and renal proximal tubular cells, incubated with high concentrations of glucose or advanced glycation end products. Mechanistically, sirtuin-1 has its renoprotective effects by modulating metabolic homeostasis and autophagy, resisting apoptosis and oxidative stress, and inhibiting inflammation through deacetylation of histones and the transcription factors p53, forkhead box group O, nuclear factor-κB, hypoxia-inducible factor-1α, and others. Furthermore, some microRNAs have been implicated in the progression of DN because they target sirtuin-1 mRNA. Several synthetic drugs and natural compounds have been identified that upregulate the expression and activity of sirtuin-1, which protects against DN. The present review will summarize advances in knowledge regarding the role of sirtuin-1 in the pathogenesis of DN. The available evidence implies that sirtuin-1 has great potential as a clinical target for the prevention and treatment of diabetes.

## Background

Diabetic nephropathy (DN), also referred to as diabetic kidney disease, occurs in 20–40% of all diabetic patients, and therefore affects hundreds of millions of people worldwide [1]. The early stages of DN are characterized by microalbuminuria, which is usually ignored. However, when this albuminuria becomes severe, it is progressive and irreversible, ultimately being associated with renal dysfunction and a high risk of cardiovascular death. Although a lot of progress has been made in the understanding of the mechanisms and appropriate treatment of DN, it remains a substantial clinical problem.

Acetylation and deacetylation regulate cell proliferation, apoptosis, autophagy, energy homeostasis, inflammation, and oxidative stress (OS). Acetylation of histone promotes transcription by opening chromatin binding sites, and acetylation of transcription factors and transcriptional coregulatory proteins regulate their transcriptional activity by modulating their subcellular localization, DNA-binding affinity, and degradation [2]. Deacetylases counteract these effects by removing acetyl groups [3]. Sirtuins are nicotinamide adenine dinucleotide (NAD+)–dependent deacetylases that function as intracellular regulators of transcriptional activity. Sirtuin-1 is the most widely expressed and extensively studied member of the sirtuin family. Six other sirtuins have been identified that have distinct biological functions associated with their distinct localization, substrate specificity, and binding partners [4]. A reduction in the NAD+/NADH ratio in the presence of excess nutrient supply results in lower renal expression of sirtuin-1 in diabetic patients and experimental models of diabetes. Accumulating evidence shows that sirtuin-1 plays a crucial role in the pathogenesis of DN [5–8]. In the present review, we discuss the role of sirtuin-1 in the pathogenesis of DN and the mechanisms involved.

## Pathogenic features and molecular mechanisms of diabetic nephropathy

DN is a chronic complication of diabetes, and its clinical and pathological characteristics require many years to develop in humans. The natural history of DN caused by type 1 diabetes mellitus (T1DM; the consequence of the autoimmune destruction of pancreatic islet beta-cells) is further divided into five stages: normoalbuminuria with hyperfiltration, microalbuminuria, macroalbuminuria, decline of the glomerular filtration rate (GFR), and the dialysis-dependent stage [9, 10]. DN caused by type 2 diabetes mellitus (T2DM; the consequence of insulin resistance (IR) and the failure of beta-cell compensation) seems to be more complex and progresses more rapidly due to the presence of IR and other disturbances, such as hypertension. The histological alterations characterizing DN can be identified in glomeruli, tubules, the interstitium, and vessels.

The glomerular defects are the most important lesions, involving a reduction in podocyte number and foot process effacement, glomerular basement membrane thickening, mesangial expansion, nodular sclerosis, and global glomerulosclerosis [11]. The reduction in podocyte number is caused by apoptosis and detachment; podocytes detachment and foot process effacement are due to cytoskeletal changes and podocyte de-differentiation. Because they are a critical component of the mechanism of glomerular filtration, the loss of and phenotypic alterations in podocytes are the principal cause of albuminuria in DN. The mesangial expansion and glomerulosclerosis result from mesangial cell proliferation, hypertrophy, phenotypic changes, and subsequently excessive mesangial matrix accumulation, which ultimately result in lower GFR. Tubular epithelial cells in diabetic kidneys can undergo hypertrophy, apoptosis, and/or transformation into mesenchymal cells. Simultaneously, the interstitium becomes infiltrated with pro-inflammatory cells and fibroblasts. The progression of tubulointerstitial fibrosis and glomerulosclerosis causes a deterioration in renal dysfunction. Furthermore, diabetes induces arteriolar hyalinosis. Thus, during the progression of DN, nearly all the cell types in the kidney demonstrate abnormalities, including proliferation, hypertrophy, de-differentiation, and apoptosis.

Several molecular mechanisms are involved in the pathogenesis of DN, namely activation of the polyol pathway during hyperglycemia, hexosamine pathway, and protein kinase C (PKC); accumulation of intracellular advanced glycation end products (AGEs); glomerular hyperfiltration; and hypertension [12]. The glucose metabolic changes result in the excessive production of free radicals, such as reactive oxygen species (ROS). ROS-induced OS causes DNA damage, specifically strand breakage and base alterations, which activate p53 and its downstream pathway to induce cell cycle arrest or apoptosis [13]. DNA damage in mitochondria results in mitochondrial dysfunction, which in turn generates more ROS. Inflammation develops as a response to OS-induced damage, which promotes repair and remodeling. This involves activation of the nuclear factor kappa B (NF-κB) pathway in renal cells, especially endothelial cells (ECs), which secrete adhesion molecules, such as intercellular adhesion molecule-1 (ICAM-1) and vascular cell adhesion protein-1 (VCAM-1), and chemokines, such as monocyte chemotactic protein-1 (MCP-1) and interleukins. These pro-inflammatory adhesion molecules and chemokines attract monocytes, macrophages, and T lymphocytes, which infiltrate kidney tissue, resulting in activation of tumor necrosis factor-α (TNF-α) signaling, and therefore the aggravation of kidney lesions and fibrosis [12].

In addition, hyperfiltration or/and metabolic changes in diabetic kidneys cause excessive oxygen consumption, which results in hypoxia and the expression of the oxygen sensor hypoxia-inducible factor-1α (HIF-1α) [14]. Activation of HIF-1 signaling also activates the vascular endothelial growth factor (VEGF) signaling pathway to promote angiogenesis. This abnormal angiogenesis induced during DN causes glomerular hypertrophy and plasma leakage, which promotes glomerulosclerosis and arteriolar hyalinosis [15, 16]. Thus, glucose metabolic changes, hemodynamic alterations, OS, inflammation, and hypoxia are the main pathophysiological features of DN. Autophagy, which is a protective mechanism that maintains intracellular homeostasis, is also impaired as a result of the activation of the pathways listed above, which aggravates renal cellular dysfunction and apoptosis.

## Evidence for the role of sirtuin-1 in humans and animal models of diabetic nephropathy

There is emerging clinical evidence to suggest that gene polymorphisms of sirtuin-1 affect patient susceptibility to DN (Table 1). Maeda et al. discovered that single nucleotide polymorphisms (SNPs) in the SIRT1 gene (encoding sirtuin-1), but not in the genes encoding other sirtuin family members (sirtuins-2–6), are associated with DN in Japanese T2DM patients [17]. Tang et al. investigated the associations between DN and SNPs in p300 and SIRT1 in Chinese patients with T2DM [18], and demonstrated that the SIRT1 rs4746720 allele C was associated with the urinary albumin/creatinine (Alb/Cre) ratio. P300 allele G and the SIRT1 TC genotype are associated with the development of DN, while the G and TT genotypes are predisposed to more severe DN. Another study of 1066 Han Chinese with T2DM showed that patients with the SIRT1 rs10823108 AA genotype had a lower risk of developing DN [8]. These associations between SIRT1 gene polymorphisms and DN suggest that sirtuin-1 is implicated in the initiation of DN.

**Table 1:** The role of sirtuin-1 in clinical studies of diabetic nephropathy

Race	Case load	Type of diabetes	The role of sirtuin-1 in DN	References
Japanese	Study 1: 747 overt proteinuria cases vs.557 controls;	T2DM	SNP rs4746720 in sirtuin-1 is significantly associated with proteinuria, and rs2236319, rs10823108, rs3818292, and rs4746720 are associated with combined phenotypes (proteinuria and ESRD).	[17]
	Study 2: 455 overt proteinuria cases vs. 965 controls;			
	Study 3: 300 end-stage renal disease cases vs.218 controls			
Chinese	DM with DN 628 cases vs. DM without DN 388 cases	T2DM	rs20551 G alleles in p300 and rs4746720 C alleles in SIRT1 correlate with an increase in ACR. P300 allele G and the sirtuin-1 TC genotype are associated with the development of DN, while the G and TT genotypes predispose to more severe DN.	[18]
Chinese	653 DM with DN vs. 413 without DN	T2DM	SIRT1 rs10823108 AA genotype is associated with a decreased risk of DN.	[8]
Chinese	495 DM patients:	T2DM	Serum Sirtuin-1 levels of diabetes patients are significantly lower than those in the control group, and decrease with the increase in ACR.	[19]
	Normoalbuminuric group (ACR < 30 mg/g, *n* = 186)			
	Microalbuminuric group (ACR 30–300 mg/g, *n* = 169)			
	Macroalbuminuric group (ACR > 300 mg/g, *n* = 140)			

*SNP*, single nucleotide polymorphism; *DM*, diabetes mellitus; *DN*, diabetic nephropathy; *ACR*, urinary albumin to creatinine ratio

A number of models of diabetes have been used to evaluate the renoprotective effects of sirtuin-1, including both T1DM [20, 21] and T2DM models [22, 23], such as the db/db diabetic mouse, streptozotocin (STZ) alone diabetic mouse/rat model, or a combination of STZ- and high-fat diet–induced diabetic mouse/rat models. To identify the role of sirtuin-1 in the kidney, Chuang et al. generated mice with an 80% reduction in sirtuin-1 expression [24], which showed no defect in glomerular function until they were rendered diabetic, when they demonstrated severe albuminuria and mitochondrial dysfunction. In addition, Hasegawa et al. used proximal tubule–specific sirtuin-1 transgenic and sirtuin-1 knockout mice to reveal that sirtuin-1 protects against albuminuria in diabetes [25]. Finally, podocyte-specific knockout of sirtuin-1 reduced the quality and quantity of podocytes and worsened albuminuria in diabetic mice [26].

In vitro, renoprotective effects of sirtuin-1 are studied in primary renal parenchymal cells and cell lines in the presence of high concentrations of glucose (HG) or AGEs.

### Podocytes

Apoptosis and the detachment of podocytes, and foot process effacement, cause albuminuria in DN. Multiple studies show that sirtuin-1 is necessary for the maintenance of cytoskeletal integrity and the survival of podocytes [27, 28]. A model of non-diabetic podocyte injury (induced by a nephrotoxic serum) revealed that sirtuin-1 can deacetylate cortactin, which is crucial for the maintenance of the actin cytoskeleton and the structure of the slit diaphragm between podocyte processes [27]. Treatment with AGE-modified bovine serum albumin downregulated sirtuin-1 in cultured podocytes, which increased the acetylation of forkhead box group O (FoxO)4, resulting in apoptosis because of greater expression of the pro-apoptotic gene BCL2 like 11 [29]. Hasegawa et al. referred to “proximal tubule-podocyte communication,” by which sirtuin-1 in tubules downregulates claudin-1 expression in podocytes to protect against diabetes-induced albuminuria [25, 30]. Claudin-1 belongs to the claudin family of proteins that constitutes the tight junction. High levels of claudin-1 have been reported to be associated with podocyte effacement and albuminuria. Sirtuin-1 downregulates the expression of claudin-1 by deacetylating histone H3 and H4. Conversely, diabetes-induced upregulation of claudin-1 in podocytes causes slit diaphragm-tight junction transition and consequently proteinuria [31]. The potential mechanism of this proximal tubule-podocyte communication will be discussed below.

### Glomerular mesangial cells

Glomerular mesangial cells (GMCs) secrete numerous cytokines under diabetic conditions, including transforming growth factor-β1 (TGF-β1), leading to the expansion of the mesangial area and glomerular sclerosis. Under these conditions, a reduction in sirtuin-1 of GMCs has been observed, which leads to inflammation and fibrosis [32]. Normally, sirtuin-1 blocks the activation of pro-hypertrophic Akt signaling, as well as augmenting the activity of anti-hypertrophic AMP-activated protein kinase (AMPK) signaling in GMCs [33]. However, sirtuin-1 protein levels and deacetylase activity decline in a dose- and time-dependent manner in GMCs cultured with AGEs [34]. Furthermore, in a rat glomerular mesangial cell line that was exposed to HG for 72–144 h, the expression of sirtuin-1 decreased, while expression of the renal pro-fibrotic factors vimentin and fibronectin (FN) was induced [35].

### Renal ECs

Although studies regarding the role of sirtuin-1 in diabetic endothelial injury have been few in number, they do provide evidence that changes in sirtuin-1 expression in ECs also involve DN. Mice with an endothelium-specific deletion of sirtuin-1 show peritubular capillary rarefaction and fibrosis, due to activation of notch-1 signaling [36]. Moreover, the cleavage of sirtuin-1 by cathepsin is involved in stress-induced premature senescence in ECs [37]. Dermal-derived human microvascular ECs incubated in HG medium show early senescence and develop an irregular and hypertrophic phenotype, associated with lower sirtuin-1 mRNA expression [38]. In addition, in human glomerular ECs exposed to HG, AMPK phosphorylation (pAMPK) and sirtuin-1 expression were lower and there was more apoptosis [39].

### Proximal tubular cells

Lower sirtuin-1 expression was observed in proximal tubular cells (PTCs), which are fragile parenchymal cells, under HG conditions. Fu et al. reported that the mRNA and protein expression of sirtuin-1 by PTCs incubated in HG decreased to 19% and 36% of the control level, respectively [40]. Xue et al. showed similar time-dependent reductions in HK-2 cells (a PTC cell line) [41]. Studies by Hasegawa et al. revealed that sirtuin-1 expression in PTCs affects glomerular function by influencing podocytes. They found that sirtuin-1 expression in PTCs is low prior to the appearance of albuminuria in STZ-induced or db/db diabetic mice. PTC-specific knockout of sirtuin-1 causes albuminuria in non-diabetic mice and aggravates the albuminuria of diabetic mice. Furthermore, PTC-specific sirtuin-1 transgenic mice are protected against DN [25]. They called this phenomenon “proximal tubule-podocyte communication” and proposed that HG stress triggers a decline in sirtuin-1 expression in proximal tubules, leading to the release of the humoral mediator nicotinamide mononucleotide (NMN), which causes an increase in claudin-1 expression in podocytes [30]. NMN is a NAD+ intermediate, which is found at a lower concentration in the medium of PTCs incubated in HG than in that of PTCs incubated with a normal concentration of glucose. When the medium from PTCs incubated in HG was used to culture podocytes, the podocytes exhibited downregulation of sirtuin-1 and upregulation of claudin-1, whereas the levels of sirtuin-1 and claudin-1 did not change when the podocytes were incubated in HG medium alone. These findings indicate that some factors (NMN, not HG) in the conditioned medium from HG-incubated PTCs affect the expression of sirtuin-1 and claudin-1 in podocytes [25]. Finally, a fluorescence-labeling technique showed that NMN could be released by PTCs and affect podocytes [30].

## Roles of sirtuin-1 in signaling

### AMPK/sirtuin-1/PGC-1α signaling

**Fig. 1:**
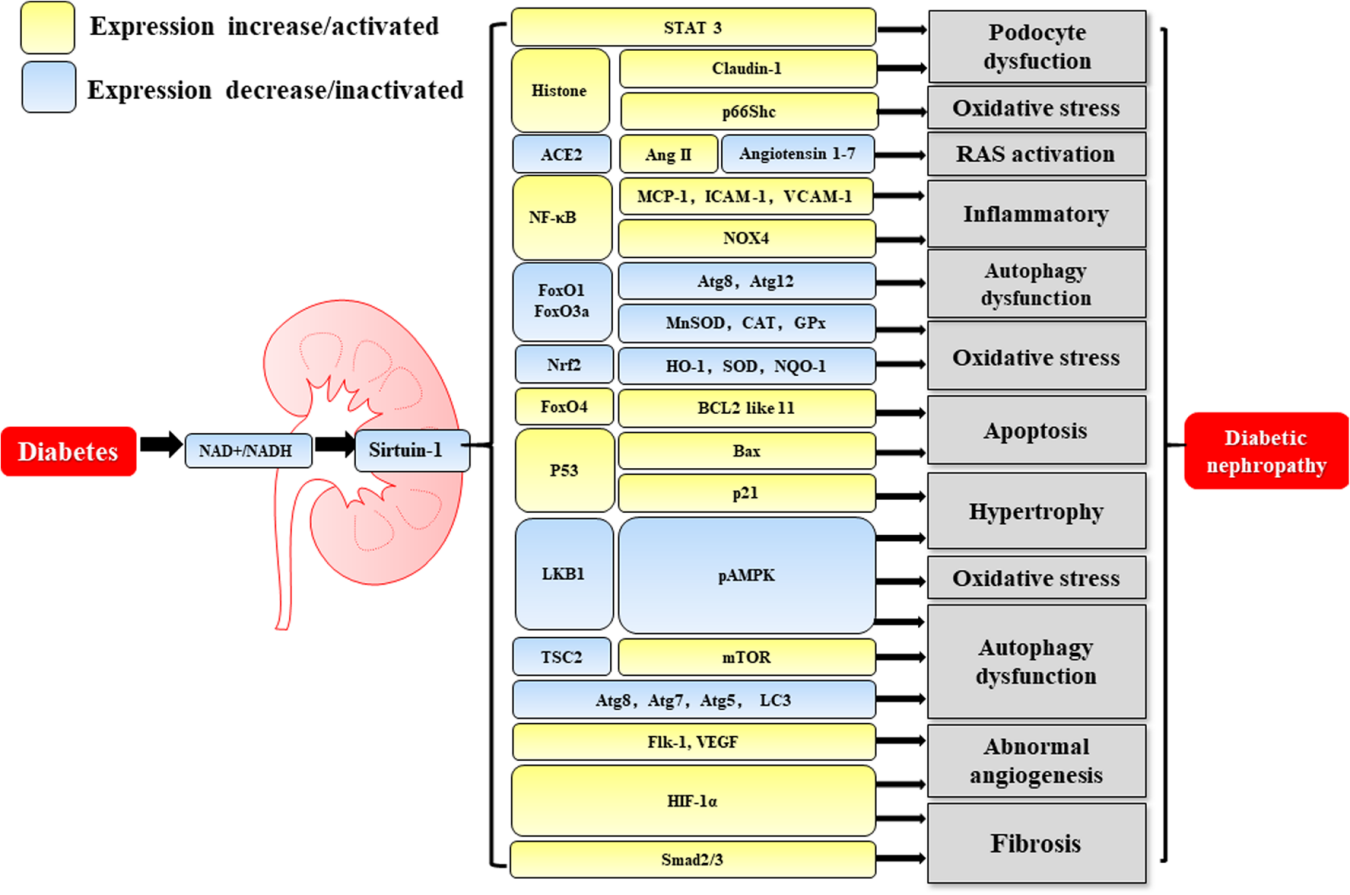
The molecular mechanisms of sirtuin-1 involvement in diabetic nephropathy. The changes in glucose metabolism in diabetes are associated with greater production of NADH and a reduction in the NAD+/NADH ratio, resulting in lower expression of sirtuin-1. Downregulation of sirtuin-1 causes greater acetylation of histones and several crucial transcription factors, such as STAT3, NF-κB, FoxO4, p53, HIF-1α, and Smad2/3, which increases their expression levels and transcriptional activation activities. Activation of STAT3 and upregulation of claudin-1 result in podocyte dysfunction. Acetylation of p66Shc facilitates its phosphorylation and translocation to the mitochondria, where it promotes hydrogen peroxide production. Activation of NF-κB signaling promotes the expression of its pro-inflammatory downstream effectors MCP-1, ICAM-1, VCAM-1, and NOX4. Acetylation of FoxO4 promotes expression of the pro-apoptotic gene BCL2 like 11, activating apoptosis. Acetylation of p53 stabilizes and activates it, resulting in target gene transcription, including that of p21 and Bax, inducing cell cycle arrest and apoptosis. Furthermore, the activation of Smad2/3 and HIF-1α induces fibrosis. HIF-1α and Flk-1 activate the VEGF pathway, causing abnormal angiogenesis. Lower expression of sirtuin-1 also leads to lower expression and/or inactivation of ACE2, FoxO1, FoxO3a, Nrf2, LKB1, TSC2, Atg8, Atg7, Atg5, and LC3, either directly (increase in acetylation) or indirectly. The inactivation of ACE2 removes its regulatory effect on Ang II and activates RAS. The inactivation of Nrf2 and the lower activity of FoxO1 and FoxO3a inhibit the expression of anti-oxidants such as Mn-SOD, CAT, GPs, HO-1, SOD, and NQO-1, which aggravates oxidative stress and mitochondrial dysfunction. The inactivation of LKB1 results in downregulation of the AMPK/PGC-1 pathway, which impairs autophagy and mitochondrial function, and promotes hypertrophy. As an inhibitor of the mTOR pathway, inactivation of TSC2 promotes activation of the mTOR pathway, which inhibits autophagy. Lower expression of Atg8, Atg7, Atg5, and LC3 impairs autophagy. Metabolic disturbance, oxidative stress, inflammation, impaired autophagy, hypoxia, abnormal angiogenesis, apoptosis, fibrogenesis, and activation of the RAS combine to cause the kidney lesions in diabetes

Under diabetic conditions, the downregulation of AMPK/sirtuin-1/PGC-1α signaling induces hypertrophy, OS, and mitochondrial and autophagy dysfunction, all which promote the development of DN (Fig. 1). Both AMPK and sirtuin-1 have been identified as intracellular energy sensors, detecting and responding to AMP/ATP and NAD+/NADH ratios, respectively, and therefore being activated under conditions of energy depletion and deactivated in diabetes [42–44]. However, AMPK and sirtuin-1 also regulate one another’s activity [42]. Sirtuin-1 deacetylates lysine residues in liver kinase B1 (LKB1), promoting its migration from the nucleus to the cytoplasm, where it can catalyze the phosphorylation and activation of AMPK [45]. Concurrently, AMPK activates downstream signaling in a sirtuin-1–dependent manner [46]. In addition, AMPK upregulates sirtuin-1 by increasing cellular NAD+ levels [47]. Finally, Fulco et al. showed that glucose restriction–induced activation of AMPK increases sirtuin-1 activity by promoting transcription of the NAD+ biosynthetic enzyme nicotinamide phosphoribosyltransferase (Nampt) [48].

Under diabetic conditions, pAMPK was lower in glomeruli and tubules, implying the inactivation of AMPK [49, 50]. Previous studies showed that calorie restriction (CR) has beneficial effects on DN via AMPK and sirtuin-1 [51]. The levels of pAMPK and sirtuin-1 are lower in diabetic kidney, but the phosphodiesterase type 4 (PDE4) inhibitor roflumilast mimics the effects of CR to restore the levels of pAMPK/sirtuin-1 and alleviate DN [52]. AMPK and sirtuin-1 share peroxisome proliferator–activated receptor (PPAR)-γ coactivator 1α (PGC-1α) as a target, which is a transcriptional coactivator that modulates metabolic homeostasis [53], and mitochondrial biogenesis and function [54]. It has been suggested that the phosphorylation of PGC-1α by AMPK makes it more susceptible to deacetylation by sirtuin-1 [47] and enhances its ability to activate its own promoter [55].

Adiponectin is an adipokine that has protective anti-oxidant and anti-inflammatory effects, but its expression is downregulated in IR, obesity, and T2DM. The receptors of adiponectin include adiponectin receptor (AdipoR)1 and AdipoR2, which activate AMPK and PPARα. Resveratrol, an activator of sirtuin-1, promotes the phosphorylation of AMPK and the activation of sirtuin-1/PGC-1α signaling, which prevents apoptosis and OS, and thereby ameliorates DN in db/db mice [56]. In addition, Park et al. reported that HG-induced OS and apoptosis in cultured glomerular ECs are prevented by resveratrol treatment, which activates AMPK/sirtuin-1/PGC-1α and PPARα signaling by increasing the expression of AdipoR1 and AdipoR2 [39].

Furthermore, several natural substances have been identified to ameliorate DN by activating AMPK/sirtuin-1/PGC-1α signaling. In vitro, glycyrrhizic acid attenuates cell proliferation and TGF-β1 overexpression, and ameliorates the low expression and activity of AMPK, sirtuin-1, and manganese superoxide dismutase (Mn-SOD) induced by HG in renal tubular cells [57]. In vivo, glycyrrhizic acid ameliorates DN by inhibiting ROS and activating AMPK/sirtuin-1/PGC-1α signaling in db/db mice [22]. Cai et al. reported that grape seed procyanidin B2 upregulated the expression of AMPK, sirtuin-1, and PGC-1α in podocytes [58], while Bao et al. showed that grape seed proanthocyanidin prevents diabetes-induced OS and mitochondrial dysfunction in podocytes by activating AMPK/sirtuin-1/PGC-1α signaling [59].

### Sirtuin-1 and mammalian target of rapamycin

Decreases in sirtuin-1 expression alleviate suppression of the mTOR pathway, which promotes HG-induced renal cell autophagy dysfunction and senescence. Mammalian target of rapamycin (mTOR) is a serine/threonine protein kinase and component of the mTOR complex, which is a key modulator of cellular proliferation, autophagy, and lipid metabolism. Tuberous sclerosis complex (TSC)1 and TSC2 are inhibitors of the mTOR signaling pathway, and it is reported that the negative regulation of mTOR signaling by sirtuin-1 is TSC2-dependent. Immunoprecipitation indicated that sirtuin-1 interacts with TSC2, but the exact mechanism is uncertain [60]. HG induces the upregulation of mTOR and downregulation of sirtuin-1 in MCs [61], while rapamycin, an inhibitor of mTORC1, inhibits the downstream pathway and arrests HG-induced MC senescence. However, the silencing of sirtuin-1 completely inhibits the effects of rapamycin in HG-treated MCs. These findings suggest that sirtuin-1 regulates the mTOR pathway, which mediates the induction of MC senescence by HG [61]. Another study confirmed that rapamycin partially inhibits mTORC1 and restores sirtuin-1 activity in palmitate-treated podocytes [62].

### Sirtuin-1 and the Keap1/Nrf2/ARE pathway

The activation of the nuclear factor erythroid 2–related factor 2 (Nrf2)/anti-oxidant response element (ARE) pathway is suppressed in DN due to low sirtuin-1 levels, which in turn decreases anti-oxidant capacity. Accumulation of AGEs in diabetic kidney stimulates the generation of ROS, causing OS and promoting the progression of DN. ROS/anti-oxidant homeostasis is disturbed when the production of ROS exceeds the production of anti-oxidants, and diabetic complications result from OS due both to excessive production of ROS and a reduction in anti-oxidant capacity [13].

The Nrf2 is a transcription factor that binds to the ARE of DNA and switches on its target genes, including NAD(P)H quinone dehydrogenase 1 (NQO-1), heme oxygenase 1 (HO-1), and superoxide dismutase (SOD). Kelch-like ECH–associated protein 1 (Keap1) acts as a negative regulator of Nrf2 by binding it, blocking its transfer to the nucleus, and promoting its degradation. The Nrf2/ARE pathway is a critical cellular anti-oxidant mechanism that is inactivated during chronic OS, including that associated with diabetes, and sirtuin-1 has a significant anti-oxidant effect because it activates the Nrf2/ARE pathway [63, 64]. Previous studies demonstrated cross-talk between sirtuin-1 and the Keap1/Nrf2/ARE pathway: sirtuin-1 enhances the activity of the Keap1/Nrf2/ARE pathway by decreasing Keap1 expression, deacetylating and reducing the ubiquitination of Nrf2, and promoting ARE-binding ability in AGE-treated GMCs [65]. In addition, Huang et al. reported that polydatin, a glucoside of resveratrol, ameliorates AGE-induced FN and TGF-β1 expression by activating the sirtuin-1/Nrf2/ARE pathway in rat GMCs [64]. Zhang et al. reported that paeonol, an extract of *Cortex moutan*, delayed the progression of fibrosis by activating sirtuin-1 and therefore the Nrf2 pathway in DN [66]. Ling Zhou et al. found that HG decreased sirtuin-1 activity in HK-2 cells, enhanced Keap1 expression, and promoted the ubiquitination and degradation of Nrf2 via a pathway involving NF-κB and microRNA (miR)-29 [67]. However, Nrf2 also positively regulates the protein expression of sirtuin-1 [65], and the Nrf2 activator tBHQ increases the expression and deacetylase activity of sirtuin-1, while small interfering (si)RNAs targeting Nrf2 reduce sirtuin-1 expression and activity, leading to greater expression of FN and TGF-β1 in GMCs [65]. Although the underlying mechanism of these effects is unknown, some studies show that Nrf2 promotes sirtuin-1 expression by negatively regulating p53 [68].

### Sirtuin-1 and p66Shc

Diabetes-induced downregulation of sirtuin-1 increases OS in mitochondria by promoting histone H3 and p66Shc acetylation. Mitochondria are the primary source of the excess ROS [13]. The adaptor protein 66 kDa Src homology 2 domain–containing protein (p66Shc) is a crucial regulator of mitochondrial ROS production and contributes to DN by promoting OS [69–71]. p66Shc mainly expresses in the renal tubular cells and glomeruli and is a direct target of sirtuin-1. A recent study indicates that HG-induced downregulation of sirtuin-1 promotes the expression of p66Shc by increasing histone H3 and p66shc acetylation [72]. Histone H3 acetylation promotes transcription of p66Shc. The acetylation of p66Shc facilitates its phosphorylation and translocation to mitochondria, where it promotes hydrogen peroxide production [69, 73, 74]. In addition, diabetes, HG, and palmitate stimulate p66Shc, which activates a redox mechanism that upregulates miR-34a expression, targeting sirtuin-1 for degradation, and causing endothelial dysfunction. These effects are prevented when p66shc expression is silenced, while overexpression of p66Shc enhances HG and palmitate-induced miR-34a expression, thus reducing that of sirtuin-1 [75].

### Sirtuin-1 and HIF-1

Sirtuin-1 deficiency under diabetic condition leads to activation of HIF-1, which results in abnormal angiogenesis and fibrosis in the kidney. Recent studies demonstrated that hypoxia is also involved in the pathogenesis of DN [14]. Hyperfiltration or/and metabolic changes in diabetic kidneys cause excessive oxygen consumption, resulting in hypoxia and expression of the oxygen sensor HIF-1. HIF-1 is composed of a functional α-subunit and a constitutive β-subunit and promotes epithelial-to-mesenchymal transition (EMT) in vitro, while renal epithelial knockout of HIF-1α prevents tubulointerstitial fibrosis [76]. HIF-1α is a downstream target of sirtuin-1, and during hypoxia downregulation of sirtuin-1 leads to greater acetylation and activation of HIF-1α. When sirtuin-1 is activated by resveratrol, HIF-1α is deacetylated and inactivated, which prevents HG-induced expression of connective tissue growth factor (CTGF), endothelin-1, FN, TGF-β1, and VEGF [32].

### Sirtuin-1 and NF-κB

Diabetes-induced downregulation of sirtuin-1 leads to activation of NF-κB signaling, which promotes the activation of downstream pro-inflammatory factors and inhibits anti-oxidative stress Nrf2/ARE pathway involved in the development of DN. NF-κB is a transcription factor that governs the expression of genes involved in inflammation. The canonical pathway of NF-κB activation involves the p65 and p50 subunits [77], and it is now known that sirtuin-1 can deacetylate the p65 subunit of NF-κB and inhibit NF-κB pro-inflammatory signaling and the downstream production of MCP-1, ICAM-1, and VCAM-1 [77–79]. MCP-1 exerts pro-inflammatory effects by regulating the migration and infiltration of monocytes/macrophages, a process that could thus be regulated by a sirtuin-1/NF-κB p65 pathway [80]. Podocyte-specific ablation of sirtuin-1 in db/db mice results in severe proteinuria and kidney injury, which is accompanied by greater acetylation of p65 and STAT3 [26]. NADPH oxidase 4 (NOX4) expression is higher in DN, while podocyte-specific knockout of NOX4 attenuates DN. Previous studies suggest that NF-κB directly regulates NOX4 expression by binding to its promoter, and sirtuin-1 could therefore ameliorate inflammation in DN, at least in part through deacetylation of NF-κB and downregulation of NOX4 [81]. NF-κB could also interact with the Nrf2/ARE pathway via sirtuin-1 and miR-29. In support of this, Zhou et al. found that, in HG-incubated tubular epithelial cells, downregulation of sirtuin-1 increased the acetylation and activity of NF-κB, which directly binds to the promoter and downregulates miR-29 expression, thereby increasing the expression of Keap1 and inhibiting the Nrf2/ARE pathway [67].

### Sirtuin-1 and FOXO

Sirtuin-1 deacetylates FOXO, which changes the transcriptional activity of FOXO target genes to prevent OS, inflammation, and apoptosis in DN. FOXO belongs to the forkhead family of transcription factors, which have a conserved DNA-binding domain. In mammals, four family members have been identified: FoxO1, FoxO3a, FoxO4, and FoxO6. The expression of FoxO1 and FoxO3a is ubiquitous, while FoxO4 is highly expressed in the muscle and heart and FoxO6 is expressed in the brain [82]. Acetylation both promotes and reduces FOXO transcriptional activity and mediates various biological functions of FOXO [83].

FoxO1 has been extensively studied and shown to regulate cellular events, such as metabolism, proliferation, redox status, stress resistance, inflammation, aging, and apoptosis [84]. Sirtuin-1 reduces the acetylation of FoxO1, which enhances the DNA-binding affinity of FoxO1 [85]. The sirtuin-1/FoxO1 pathway has an anti-oxidant effect because it increases expression of Mn-SOD and catalase (CAT) [86, 87]. However, the expression of FoxO1 is lower, which is associated with the accumulation of extracellular matrix (ECM) and OS in type 1 diabetic kidney. Xu et al. found that puerarin upregulated the expression of sirtuin-1, FoxO1, and PGC-1α in renal cortex and was therefore protective against DN [87]. In addition, treatment of DN with the sirtuin-1 agonist resveratrol increased SOD activity and reduced MDA, collagen IV, and FN expression by increasing FoxO1 activity [88].

FoxO3a regulates cellular processes, including metabolism, cellular proliferation and differentiation, OS resistance, inflammation, aging, and apoptosis [84]. Sirtuin-1 deacetylates FoxO3a, which enhances FoxO3a-induced autophagy and anti-oxidant effects, while suppressing FoxO3a-induced cell death. Resveratrol deacetylates FoxO3a and attenuates the OS caused by hyperglycemia, both in vivo and in vitro. Silencing sirtuin-1 induces overexpression of acetylated FoxO3a, which aggravates the OS in HG-induced tubular epithelial cells; resveratrol treatment fails to protect against these effects [89]. Sirtuin-1 activates autophagy by deacetylating FoxO1 and FoxO3a in the nucleus [90–92], the mechanisms of which will be described below.

In addition to FoxO1 and FoxO3a, FoxO4 is deacetylated by sirtuin-1. It is involved in cellular proliferation, inflammation, aging, and apoptosis in mammals [84]. AGEs cause podocyte apoptosis by downregulating sirtuin-1 and increasing acetylation of FoxO4, which promotes expression of BCL2 like 11 [29].

### Sirtuin-1 and p53

Diabetes-induced decreases in sirtuin-1 expression involve apoptosis via activation of the p53 pathway. Apoptosis of podocytes, ECs, and tubular epithelial cells causes albuminuria and renal dysfunction in DN. Sirtuin-1 exerts anti-apoptotic effects to protect against cellular injury by deacetylating pro-apoptotic proteins. p53 is a tumor suppressor gene that is activated and induces cell cycle arrest or apoptosis under DNA-damaging conditions [93]. Acetylation stabilizes and activates p53, promoting target gene transcription, including that of p21 and Bax [93]. Sirtuin-1 negatively regulates p53 by deacetylating it at the C-terminal lysine-382 residue, and this relationship between sirtuin-1 and p53 is closely associated with aging and diabetes [93]. The sirtuin-1/p53 pathway has also been implicated in HG-induced apoptosis: study of cultured PTCs has shown that incubation in HG reduces sirtuin-1 protein expression and increases the expression of c-caspase-3 and c-PARP, and the acetylation of p53, especially when small interfering RNA against sirtuin-1 are used [94]. In addition, resveratrol prevented increases in expressions of p38 and p53, the dephosphorylation of histone H3, PTC apoptosis, and albuminuria in DN by activating sirtuin-1 [94, 95]. A recent study suggested that a p53/miR-155-5p/sirtuin-1 loop is present in the kidney of animals with DN: p53 promoted the expression of miR-155-5p, which reduced sirtuin-1 expression, in turn downregulating sirtuin-1 and disinhibiting p53 [96].

### Sirtuin-1 and autophagy-related proteins

Decreases in sirtuin-1 expression inhibit autophagy under diabetic conditions by suppressing the expression of autophagy-related proteins, FOXO and AMPK, and activating mTOR. Autophagy is a regulated process that disassembles and recycles unnecessary or dysfunctional cellular components. The process of macroautophagy, the most intensively investigated type of autophagy, includes phagophore formation and elongation, autophagosome formation, lysosome fusion, and degradation in autolysosomes [97]. Autophagy-related (Atg)5, Atg7, and Atg8 are required for the formation and elongation of the autophagosomal membrane, while microtubule-associated protein light chain 3 (LC3) and pro-autophagic Bcl2/adenovirusE1 V 19 kDa interacting protein 3 (Bnip3) are necessary for autophagy. Under starvation conditions, sirtuin-1 promotes the activity of FoxO1 and FoxO3a, which are localized to the nucleus and bind to promoter sequences of Atg8 and Atg12 [90]. Deacetylation of LC3 by sirtuin-1 promotes its nucleocytoplasmic transport and the formation of autophagosomes [98]. An increasing body of evidence indicates that a deficiency in autophagy contributes to the pathogenesis of DN [99–102]. Sirtuin-1 may restore autophagy by deacetylating FoxO1 and FoxO3 in the nucleus [90–92] and Atg5, Atg7, and Atg8 in the cytosol [103]. Activation of sirtuin-1 restores the expression of FoxO3, which positively regulates Bnip3, and thus enhances autophagy in the kidneys of db/db mice [7]. Knockdown of sirtuin-1 inhibits Atg7, Atg5, and LC3, and abolishes the resveratrol-mediated amelioration of the defect in autophagy induced by hypoxia in cultured PTCs [7].

Furthermore, both the AMPK and mTOR pathways regulate autophagy. AMPK is a positive regulator of autophagy, while the mTOR pathway is a negative regulator, activating or inhibiting UNC51-like kinase 1 complex, which is essential for the initiation of autophagy [104, 105]. Indeed, the impaired autophagy observed in DN is associated with defects in both the mTOR and AMPK pathways. Thus, sirtuin-1 might regulate autophagy via mTOR and AMPK in diabetic kidneys. Recently, a study showed that a p53/miR-155-5p/sirtuin-1 pathway regulates autophagy in PTCs incubated in HG [96], which will be discussed further below.

### Sirtuin-1 and the TGF-β1/smad pathway

Sirtuin-1 prevents diabetic renal fibrogenesis by inhibiting TGF-β1/small mothers against decapentaplegic homolog (smad) 2/3 pathway. Tubulointerstitial fibrosis and renal dysfunction are pathological features of the end stage of various kidney diseases, including DN. There is increasing evidence of the anti-fibrotic effects of sirtuin-1 in experimental models of diabetes. TGF-β1 is a pro-fibrogenic factor that is upregulated in DN; it binds to its receptor and activates the downstream mediators smad 2/3 [106, 107]. Phosphorylation and acetylation of smad2/3 enhance their activity and cause accumulation of ECM, including of collagen and FN [106–109]. However, smad2/3 have also been identified as targets of sirtuin-1. Recent studies report that TGF-β1 promotes smad2/3 acetylation, while resveratrol treatment deacetylates smad3 by activating sirtuin-1 in cultured PTs [110]. In addition, resveratrol administration abolishes TGF-β1/smad3-induced renal fibrosis in a mouse model of unilateral ureteral obstruction. In subtotally or 5/6 nephrectomized rat models, sirtuin-1 activator SRT3025 attenuated TGF-β1 overexpression, GFR decline, and proteinuria [108]. However, studies investigating whether sirtuin-1 can prevent renal fibrogenesis in DN through TGF-β1/smad2/3 deacetylation have not been reported [110]. Another study revealed reduced sirtuin-1 and increased acetylase p300, TGF-β1, and collagen I expression in HG-incubated microvascular ECs [111]. Diabetes caused high TGF-β1 expression in the kidney and albuminuria was blunted in sirtuin-1 overexpressing transgenic mice.

### Sirtuin-1 and STAT3

Signal transducer and activator of transcription3 (STAT3) are transcription factors, which are activated under diabetic conditions. The transcriptional activity of STAT3 is negatively regulated by sirtuin-1. A study by Liu et al. revealed that phosphorylation and acetylation of p65 and STAT3 were higher in the glomeruli of db/db mice than in those of db/m mice [26]. They also confirmed that acetylation of p65 and STAT3 is required for their transcriptional activity by mutating the acetylated residues. Knockout of sirtuin-1 in podocytes significantly increased acetylation of p65 and STAT3 and the mice were more susceptible to DN.

### Sirtuin-1 and VEGF mediate angiogenesis

Reduced sirtuin-1 expression results in angiopoietin 2, VEGF, and Flk-1 upregulation, which eventually leads to abnormal angiogenesis in DN. Abnormal angiogenesis occurs in the kidneys of patients and animal models with DN. Emerging evidence demonstrates that abnormal angiogenesis is associated with glomerular hypertrophy and plasma leakage, which play a pathological role in DN [112]. VEGF is a pro-angiogenic factor that binds to its receptor VEGFR-2 (KDR/Flk-1) and generates angiogenic signals by dimerization and phosphorylation of VEGFR-2 [113]. Angiopoietins are vascular growth factors, including angiopoietin 1 (effective factor) and angiopoietin 2 (antagonist of angiopoietin 1). Angiopoietin 1 and angiopoietin 2 competitively bind to their receptor tie-2 (tyrosine kinase with Ig and EGF homology domain 2). High concentrations of angiopoietin 2 and VEGF promote endothelial proliferation and angiogenesis, whereas apoptosis is induced when the upregulation of angiopoietin 2 is not paralleled by that of VEGF [114]. It is reported that VEGF, Flk-1, and angiopoietin 2 are upregulated in DN, and the upregulation of sirtuin-1 in cultured ECs reduces Flk-1 expression. In addition, resveratrol has beneficial effects in cultured podocytes and ECs by ameliorating the HG-induced expression of VEGF and Flk-1, but these effects are abolished by knocking down sirtuin-1 [115].These findings suggest that sirtuin-1 can attenuate abnormal angiogenesis in DN by modulating VEGF, Flk-1, and angiopoietin 2.

### Sirtuin-1 and the renin-angiotensin system

Downregulation of sirtuin-1 is involved in DN partly via its interacting with RAS. Inhibition of the renin-angiotensin system (RAS) benefits diabetic and DN patients, which indicates that the RAS is involved in the progression of DN. Angiotensin II (Ang II) is the principal effector of the RAS, but angiotensin-converting enzyme 2 (ACE2) counteracts the effects of Ang II by hydrolyzing Ang II to form angiotensin 1–7, which is protective against DN [116]. There is some evidence to suggest that sirtuin-1 interacts with the RAS: sirtuin-1 targets and activates the ACE2 promoter [116, 117], while Ang II regulates sirtuin-1 expression and activity. The expression of sirtuin-1 declines and the acetylation of p53 increases in a time-dependent fashion in Ang II–treated podocytes. Olmesartan, an angiotensin receptor blocker, is reported to increase sirtuin-1 activity, and to reduce p53 acetylation and p38 phosphorylation in the kidneys of diabetic db/db mice [118]. Furthermore, angiotensin 1–7 could protect against DN in db/db mice by increasing sirtuin-1 expression [116].

## Endogenous factors regulate sirtuin-1 in diabetic nephropathy

### Nampt and sirtuin-1

Metabolism of glucose and fatty acids produces NADH, which results in a reduction in the NAD+/NADH ratio under conditions of nutrient excess, such as diabetes, and is also associated with a reduction in the expression of sirtuin-1. Nampt is a key enzyme catalyzing NAD+ biosynthesis. Endogenous Nampt expression in the kidneys of STZ-induced diabetic rats was reported to be 2.36-fold higher than that of control rats [35], and immunohistochemistry demonstrated that Nampt is localized to the glomerular and tubular cells of diabetic rats [119]. In vitro, progressively higher expression of Nampt, NF-κB p65, FoxO1, and Bax, and progressively lower expression of sirtuin-1, were observed in the glomerular mesangial HBZY-1 cell line when it was exposed to HG conditions, which induced excessive expression of vimentin and FN [35].

### Heterogeneous nuclear ribonucleoprotein F and sirtuin-1

Heterogeneous nuclear ribonucleoprotein F (hnRNP F) belongs to the pre-mRNA-binding protein family and regulates gene expression at transcriptional and post-transcriptional levels. It was reported that the expression of hnRNP F, sirtuin-1, and FoxO3a was lower in human type 2 diabetic kidneys than non-diabetic kidneys. Overexpression of hnRNP F leads to higher levels of binding to the sirtuin-1 promoter, activating transcription, and leading to the attenuation of OS in PTCs, tubulointerstitial fibrosis, and apoptosis in db/db mice, because of deacetylation of Foxo3a and p53, and greater expression of CAT [120].

### MicroRNAs and sirtuin-1

**Fig. 2:**
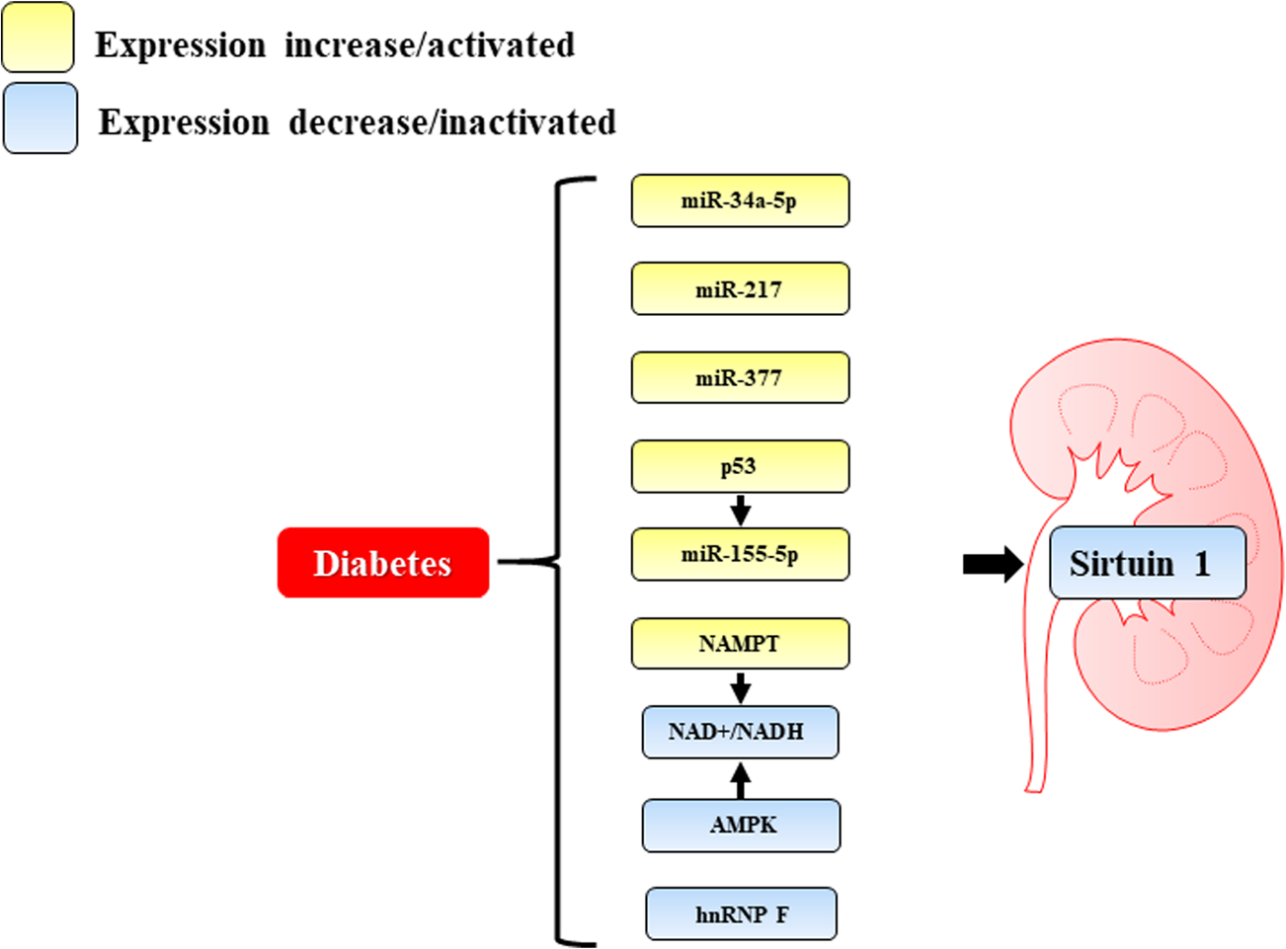
Endogenous factors regulating sirtuin-1 in diabetic nephropathy. A reduction in the NAD+/NADH ratio induced by higher Nampt expression and the metabolism of glucose and fatty acids causes a downregulation of sirtuin-1 during diabetes. Low expression of hnRNP F in diabetes downregulates sirtuin-1 transcription. High expression of miR-34a-5p, miR-377, and miR-155-5p suppresses sirtuin-1 levels by directly targeting its 3’-untranslated region. Upregulation of miR-34a-5p and miR-217 expression is negatively correlated with sirtuin-1 expression in diabetic nephropathy. Diabetes-induced DNA damage promotes the expression and activity of p53, which induces miR-155-5p expression and inhibits sirtuin-1 expression. Downregulation of AMPK decreases the NAD+ concentration and consequently the expression of sirtuin-1

miRNAs are 21–25 nucleotide–long non-coding RNAs that regulate target gene expression by binding to their 3’-untranslated regions (UTRs), causing degradation or repressing their translation. Accumulating evidence demonstrates that specific miRNAs contribute to the pathogenesis of DN by targeting sirtuin-1 mRNA (Fig. 2).

miR-34a-5p has been identified to suppress sirtuin-1 by directly targeting its 3’-UTR. When miR-34a-5p expression is high, sirtuin-1 expression falls, increasing the expression of the pro-fibrogenic factor TGF-β1, FN, and collagen type I in the kidneys of high-fat diet and STZ-induced diabetic mice [41]. It is also known that long non-coding RNAs (lncRNAs) can interact with miRNAs. 1700020I14Rik (ENSMUST00000147425) is a lncRNA that directly targets miR-34a-5p and interacts with it in an Ago2-dependent manner. Downregulation of 1700020I14Rik has been shown to promote proliferation and fibrosis via a miR-34a-5p/sirtuin-1 pathway in MCs [121].

Recently, a clinical study suggested that urinary miR-377 could be used as an early biomarker of DN in pediatric T1DM. The urinary miR-377 level is higher in T1DM patients with microalbuminuria, than in both normoalbuminuric patients and healthy controls [122]. HG triggers the upregulation of miR-377 in GMCs, which inhibits PPARγ expression and promotes plasminogen activator inhibitor-1 (PAI-1) and TGF-β1 expression [123]. miR-377 was identified to target the 3’-UTR of sirtuin-1 mRNA directly and reduces sirtuin-1 protein abundance in the adipose tissue of high-fat diet–fed mice [124]. However, further studies regarding the role of the interaction between miR-377 and sirtuin-1 in the pathogenesis and development of DN are required.

Another clinical study showed that miR-217 closely correlates with the severity of DN in patients with T2DM. Serum miR-217 levels positively correlate with the severity of albuminuria and negatively correlate with sirtuin-1 expression in patients with T2DM [19]. In addition, the study by Shao et al. showed that miR-217 expression increases in HG-treated rat GMCs. By contrast, silencing miR-217 or resveratrol treatment promotes the expression of sirtuin-1, which results in lower expression of HIF-1α and ameliorates HG-induced increases in CTGF, endothelin-1, FN, TGF-β1, and VEGF expression in rat GMCs [32].

Recently, miR-155-5p has become of interest due to its effects on renal tubule injury as part of a p53/miR-155-5p/sirtuin-1 pathway. Baker et al. measured the expression of this miRNA in the glomeruli and proximal tubules of 98 patients and discovered that miR-155-5p expression is high in the proximal tubules of individuals with DN, and in membranoproliferative glomerulonephritis and focal segmental glomerulosclerosis [125]. Bioinformatic analysis predicted the existence of binding sites of miR-155-5p on sirtuin-1 mRNA, and Wang et al. confirmed the presence of a p53/miR-155-5p/sirtuin-1 pathway in tubule cells incubated in HG medium [96].

### Ubiquitin-specific protease 22 and sirtuin-1

AGEs regulate sirtuin-1 expression by enhancing its ubiquitination and proteasome-mediated degradation. Ubiquitin-specific protease 22 (USP22) reduces the degradation of sirtuin-1 and the expression of FN and TGF-β1 in AGE-treated GMCs, whereas depletion of USP22 promotes sirtuin-1 degradation and the expression of FN and TGF-β1 in this cell model. Therefore, an AGE/USP22/sirtuin-1 pathway may be involved in the pathological progression of DN [126].

In addition to the above, caspase1, Ang II, and cyclin-dependent kinase 5 (CDK5) have been shown to regulate the level and activity of sirtuin-1 in other tissue and models, indicating that they are probably also involved in the development of DN. Caspase 1 is a member of the cysteine protease caspase family, which is activated by the “inflammasome” and required for the cleavage of multiple substrates, such as IL-1β, Il-18, and sirtuin-1; thus, it is probably also involved in atherosclerosis and DN [127, 128]. One study suggested that activation of caspase 1 decreases the level of sirtuin-1 by cleaving sirtuin-1 and promotes pro-inflammatory cytokine activation in the ECs of the aorta of ApoE−/− mice fed a high-fat diet, resulting in the activation of ECs and the initiation of vascular inflammation [127]. Knockout of caspase 1 increased sirtuin-1 levels and decreased aortic monocyte recruitment. Whether caspase-1 involves in DN via regulating sirtuin-1 is required to study. As mentioned in the section of sirtuin-1 and RAS, Ang II regulates sirtuin-1 expression and activity. A study from Huang found that Ang II activated JKN and subsequently led to sirtuin-1 degradation, which enhanced insulin-like growth factor receptor II signaling during Ang II cardiac hypertrophy and apoptosis [129]. Ang II time dependently reduced sirtuin-1 expression and induced apoptosis of cultured podocytes, which was reversed by the AT1 blocker olmesartan [118]. CDK5 is a serine/threonine kinase that phosphorylates sirtuin-1 and inhibits the anti-senescent and anti-inflammatory activity of sitruin-1 [130]. Phosphorylation of sirtuin-1 at S47 by CDK5 prevents sirtuin-1 nuclear exportation and association with the telomeric repeat–binding factor 2–interacting protein 1 in ECs. Recently, the CDK5-dependent ubiquitin-proteasome pathway was reported to mediate the degradation of sirtuin-1 in Parkinson’s disease models. Inhibition of CDK5 blocked sirtuin-1 degradation [131]. Although CDK5 contributes to podocyte apoptosis and renal tubulointerstitial fibrosis in DN [132, 133], it is unknown whether CDK5 suppression reserves DN by blocking sirtuin-1 degradation.

## Treatments targeting sirtuin-1 in diabetic nephropathy

There is a growing literature describing the therapeutic effects of synthetic drugs and natural compounds on inflammation, OS, apoptosis, and fibrosis in DN, which are exerted through the upregulation or activation of sirtuin-1.

As shown in Table 2, it is clear that several types of kidney cells are responsive to sirtuin-1 activators such as resveratrol. These findings provide a foundation for preclinical and clinical trials targeting sirtuin-1 in diabetic animals and patients with diabetes.

**Table 2:** Synthetic drugs and natural compounds that increase the expression of or activate sirtuin-1 in high glucose or advanced glycation end product–treated renal cells

Drug or natural substances	Renal cell	Mechanism of renoprotection	Pathway	Reference
Glycyrrhizic acid	HG-treated renal tubular epithelial cell line (NRK-52E)	Anti-oxidant, anti-proliferative	AMPK/sirtuin-1/PGC-1α signaling, TGF-β1, Mn-SOD	[57]
Glucagon-like peptide-1	Podocytes cultured in HG medium	Anti-apoptotic, anti-oxidant, anti-inflammatory	Sirtuin-1, IL-1, IL-6	[134]
Grape seed procyanidin B2	High-dose glucosamine-treated rat mesangial cell line (HBZY-1)	Restore mitochondrial function, anti-apoptotic, anti-oxidant	AMPK/sirtuin-1/PGC-1α	[135]
Metformin	HG-treated primary rat podocytes	Improve podocyte insulin resistance and glucose uptake, reduce glomerular filtration barrier permeability	AMPK, sirtuin-1	[136]
Olmesartan	HG-treated conditionally immortalized mouse podocytes	Anti-apoptotic	Angiotensin II/p38/sirtuin-1	[118]
Probucol	HG-treated human proximal tubular epithelial cells (HK-2)	Anti-oxidant, anti-fibrotic	p66Shc, AMPK/sirtuin-1/AcH3	[137]
Puerarin (active compound of radix puerariae)	HG-treated conditionally immortalized murine podocytes	Anti-oxidant	Sirtuin-1, NF-κB, NOX4	[81]
Polydatin (glucoside of resveratrol)	Advanced glycation end product–treated glomerular mesangial cells	Anti-oxidant	Sirtuin-1/Nrf2/ARE	[64]
*Panax notoginseng* saponins	HG-treated rat mesangial cells	Anti-inflammatory, anti-oxidant, anti-fibrotic	Sirtuin-1/NF-κB, PAI-1, TGF-β1, MCP-1, SOD	[80]
Resveratrol	HG-treated human endothelial cells	Counteract the other pro-atherosclerotic effects, downregulate endothelial nitric oxide synthase	Sirtuin-1	[138]
	HG-treated primary rat mesangial cells	Anti-senescent	mTOR, sirtuin-1	[61]
	HG-treated primary rat mesangial cells	Anti-oxidant, restore mitochondrial function	Sirtuin-1, Mn-SOD	[63]
	HG-treated conditionally immortalized mouse podocytes; HG-treated immortalized mouse endothelial cell line	Suppress VEGF expression and secretion in podocytes, suppress Flk-1 expression in glomerular endothelial cells, ameliorate hyperpermeability and cellular junction disruption	Sirtuin-1, VEGF, Flk-1	[115]
	Advanced glycation end product–treated rat primary glomerular mesangial cells	Anti-oxidant, anti-fibrotic	Sirtuin-1, Nrf2/ARE, TGF-β1	[34]
	HG-treated NMS2 mesangial cells	Anti-oxidant, anti-apoptotic	AMPK/sirtuin-1/PGC-1α	[56]
	HG-treated human kidney epithelial (HK-2) cells	Anti-oxidant	Sirtuin-1/FOXO3a	[89]
Shenkang injection (composed of Radix Astragali, Rhubarb, Astragalus, Safflower, and Salvia)	HG-treated primary renal proximal tubular epithelial cells	Anti-senescent, anti-oxidant	mTOR, p66Shc, sirtuin-1, PPARγ, P16^INK4^, cyclin D1, SOD	[40]
Theobromine	HG-treated immortalized human mesangial cells	Anti-fibrotic	NOX4, AMPK, sirtuin-1/TGF-β	[139]
Tetrahydroxystilbene glucoside (active component of *Polygonum multiflorum* Thunb)	HG-treated rat mesangial cell line (HBZY-1)	Anti-oxidant	Sirtuin-1/TGF-β1, COX-2	[140]

A list of preclinical studies of treatments for DN that target sirtuin-1 is shown in Table 3. These have shown the following effects: (1) a reduction in the urinary Alb/Cre ratio or 24 h urine albumin; (2) an amelioration of renal histopathology; (3) reductions in markers of OS, inflammation, and apoptosis; (4) an improvement in autophagy; and/or (5) prevention of fibrogenesis. Furthermore, we showed that fenofibrate, a PPARα agonist, can stimulate fibroblast growth factor 21/sirtuin-1–dependent autophagy, which can prevent T1DM-induced cardiac damage [145]. We also found that it protects against T1DM-induced nephropathy by activating fibroblast growth factor 21 and Nrf2 pathways, although sirtuin-1 was not implicated in this study [146]. Thus, whether the renoprotective effects of fenofibrate in T1DM are due to the upregulation of sirtuin-1 remains unclear.

**Table 3:** Synthetic drugs and natural compounds identified as regulators of sirtuin-1 in preclinical studies of diabetic nephropathy in animal models

Substance	Animal model	Mechanism of renoprotection	Pathway	Reference
3,5-Diiodo-L-thyronine	T1DM: STZ-induced diabetic rat	Prevent decrease in sirtuin-1 expression and activity; anti-fibrotic transforming growth factor-β1 expression, fibronectin and type IV collagen	Sirtuin-1/NF-κB	[141]
*Allium sativum* (garlic)	T1DM: STZ-induced diabetic rat T2DM: STZ + niacinamide–induced diabetic rat	Increase sirtuin-1 and sirtuin-2 gene expression in kidney	Sirtuin-1; sirtuin-2	[21]
Glycyrrhizic acid	T2DM: db/db mouse	Anti-oxidant, anti-fibrotic	AMPK/sirtuin-1/PGC-1α signaling	[22]
Grape seed proanthocyanidin extracts	T2DM: high-carbohydrate/high-fat diet and STZ-induced diabetic rat	Restore mitochondrial function, anti-apoptotic, anti-oxidant, increase nephrin and podocalyxin	AMPK/sirtuin-1/PGC-1α	[59]
Hesperidin and quercetin	T1DM: STZ-induced diabetic rat	Anti-oxidant	NF-κB, sirtuin-1, SOD, CAT	[142]
INT-777 (G protein–coupled bile acid receptor TGR5 agonist)	T2DM: db/db mouse	Increase renal mitochondrial biogenesis, decrease oxidative stress, increase fatty acid beta-oxidation	Sirtuin-1, sirtuin-3, Nrf1, SOD2	[143]
Olmesartan	T2DM: db/db mice	Anti-apoptotic, suppress p38 phosphorylation	Angiotensin II/p38/sirtuin-1	[118]
Probucol	T2DM: high-fat, high-cholesterol Western diet and STZ-induced diabetic mice	Anti-oxidant, anti-fibrotic	p66Shc, AMPK/sirtuin-1/AcH3	[137]
Puerarin (active compound of Radix Puerariae)	STZ-induced diabetes in endothelial nitric oxide synthase–null (eNOS−/−) mouse	Anti-oxidant	NF-κB, NOX4	[81]
	T1DM: STZ-induced diabetic mouse	Anti-oxidant, anti-inflammatory	Sirtuin-1/FOXO1, TNF-α, NF-κB, IL-6	[87]
*Panax notoginseng* saponins	T1DM: alloxan-induced rat	Anti-inflammatory, anti-oxidant, anti-fibrotic	Sirtuin-1/NF-κB, PAI-1, TGF-β1, MCP-1, SOD	[80]
Resveratrol	T1DM: STZ-induced diabetic rat	Anti-oxidant, prevent dephosphorylation of histone H3, reduce the expression of p38 and p53	Sirtuin, p53, p38	[95]
	T1DM: STZ-induced diabetic rat	Anti-oxidant	Sirtuin-1/FOXO1	[88]
	T1DM: STZ-induced diabetic rat	Modulate angiogenesis	Sirtuin-1, VEGF, Flk-1, Tie-2	[115]
	T1DM: STZ-induced diabetic rat	Anti-oxidant, anti-fibrotic	Sirtuin-1, Nrf2/ARE, TGF-β1, FN	[34]
	T2DM: db/db mouse	Prevent renal lipotoxicity and glucotoxicity, anti-oxidant, anti-apoptotic	AMPK/sirtuin-1/PGC-1α	[56]
	T2DM: STZ-induced diabetic rat	Anti-inflammatory; enhance autophagy	NAD+/sirtuin-1, TNF-α, IL-6, IL-1β, IL-10	[7]
	T1DM: STZ-induced diabetic rat	Anti-oxidant	Sirtuin-1/FoxO3a	[89]
Resveratrol and rosuvastatin	T2DM: STZ + niacinamide–induced diabetic rat	Anti-oxidant, anti-fibrotic	TGF-β1, NF-κB/p65, Nrf2, sirtuin-1, FoxO1	[144]
Roflumilast	T1DM: STZ-induced diabetic rat	Anti-oxidant, anti-fibrotic, anti-apoptotic	AMPK/sirtuin-1, FoxO1, HO-1	[52]
Theobromine	Spontaneously hypertensive rat treated with STZ	Anti-fibrotic	NOX4, AMPK, sirtuin-1/TGF-β	[139]
Tetrahydroxystilbene glucoside (active component extract of *Polygonum multiflorum* Thunb)	T1DM: STZ-induced diabetic rat	Anti-oxidant	Sirtuin-1/TGF-β1, COX-2	[140]

A number of substances and plant extracts that have been found to restore sirtuin-1 activity and have renoprotective effects in preclinical models of DN have been prescribed clinically. Of these, resveratrol is one of the most extensively studied sirtuin-1 activators. However, although the protective effects of resveratrol have been shown in vitro and in vivo, its clinical benefits are controversial [147]. A randomized double-blind placebo-controlled trial revealed that 6 months of low-dose or high-dose resveratrol supplementation did not improve arterial pressure, blood glucose, uric acid, adiponectin, or IL-6 in patients with T2DM [148]. By contrast, a study of 66 patients with T2DM showed that resveratrol (1 g/day for 45 days) improved systolic blood pressure, blood glucose, and IR, but not kidney function [149]. This study used creatinine, rather than urinary Alb/Cre, as an index of kidney function, which is probably why a renoprotective effect was not identified. Another randomized double-blind clinical trial evaluated the effects of resveratrol on albuminuria in DN. The 60 patients enrolled, who had DN and albuminuria, were divided into two groups: resveratrol- (500 mg/day) and losartan (an angiotensin receptor blocker, 12.5 mg/day)-treated, and placebo- and losartan (12.5 mg/day)-treated. After 90-day treatment, the urinary Alb/Cre ratio was significantly lower in the resveratrol group, although GFR and serum creatinine were not different. Although sirtuin-1 was not measured in this study, serum anti-oxidant enzymes, such as SOD, CAT, and glutathione peroxidase, and nitric oxide, were significantly higher in the resveratrol group [150].

Grape seed extracts have been shown to protect against diabetes-induced kidney lesions by activating sirtuin-1 in renal cell lines and animal models. A double-blind randomized controlled trial demonstrated that grape seed extracts could benefit T2DM patients with high cardiovascular risk by ameliorating inflammation and OS [151], but renoprotective effects and sirtuin-1 levels were not evaluated in this trial.

Inhibition of the RAS benefits patients with DN. Olmesartan, an angiotensin receptor blocker, has been shown to prevent microalbuminuria in T2DM patients [152]. In vitro and in vivo studies showed that one of its renoprotective effects is to reduce podocyte apoptosis by increasing sirtuin-1 expression [118], although the mechanism has not been identified in humans.

So far, sirtuin-1 activators, such as resveratrol, have not been shown definitively to have beneficial effects on DN in clinical trials, although they have been shown to have renoprotective effects in preclinical studies. DN takes several weeks to develop in animal models but many years to develop in humans; thus, the processes involved in the development of DN in human are probably more complicated than those in animal models. This might explain, at least partially, the differences in the effects of sirtuin-1 activators between clinical and preclinical studies. In addition, DN is generally progressive and irreversible at the time it is diagnosed. Therefore, when treatment is initiated will have a profound effect on the therapeutic outcomes of sirtuin-1 activator treatments in DN patients.

## Conclusions and perspectives

Metabolic disturbance, OS, inflammation, impairs autophagy, hypoxia, abnormal angiogenesis, apoptosis, and activation of the RAS result in kidney lesions in diabetes. The deacetylase sirtuin-1 is involved in all of these aspects of the pathogenesis of DN. Downregulation of sirtuin-1 in diabetes increases the acetylation of histones and that of crucial transcription factors, including p53, FoxO, NF-κB, and Nrf2, which are involved in numerous feedback loops and networks that promote the development of DN. Its key role in DN makes sirtuin-1 a target for preventive and therapeutic purposes. Here, we summarized the synthetic drugs and natural compounds that are used in the treatment of DN and which target sirtuin-1. In vitro studies have identified the molecular effects of these substances in various types of renal cells, and preclinical studies have shown protective effects against DN and on sirtuin-1 and its downstream target proteins. Although some clinical trials demonstrated that sirtuin-1 activators, such as resveratrol, benefit patients with T2DM and microalbuminuria, others did not show protective effects, possibly due to differences in dose, disease stage, treatment duration, and the characteristics of the patients studied. Because the regulation and effects of sirtuin-1 are complex, further investigation of its molecular interactions, such as with miRNAs and lncRNAs, which may underpin its protective effects, is required. In addition, well-designed clinical trials in patients with T2DM and T1DM are required to assess the renoprotective effects of substances that have been shown to have beneficial effects exerted via sirtuin-1 in vitro and in vivo.

## Abbreviations

ACE2: angiotensin-converting enzyme 2
AdipoR: adiponectin receptor
AGE: advanced glycation end product
AMPK: AMP-dependent protein kinase
Ang II: angiotensin II
ARE: anti-oxidant response element
Atg: autophagy-related gene
Bnip3: Bcl2/adenovirusE1 V 19 kDa interacting protein 3
CAT: catalase
CDK5: cyclin-dependent kinase 5
CR: calorie restriction
CTGF: connective tissue growth factor
DN: diabetic nephropathy
ECM: extracellular matrix
EC: endothelial cell
EMT: epithelial-to-mesenchymal transition
FN: fibronectin
FOXO: forkhead box group O
GFR: glomerular filtration rate
GMC: glomerular mesangial cell
HG: high glucose
HIF-1α: hypoxia-inducible factor-1α
hnRNP F: heterogeneous nuclear ribonucleoprotein F
HO-1: heme oxygenase 1
ICAM-1: intercellular adhesion molecule-1
IR: insulin resistance
Keap1: Kelch-like ECH–associated protein 1
LC3: light chain 3
LincRNA: long intergenic non-coding RNA
LKB1: liver kinase B1
lncRNA: long non-coding RNA
MCP-1: monocyte chemotactic protein-1
miRNA: microRNA
Mn-SOD: manganese superoxide dismutase
NAD+: nicotinamide adenine dinucleotide
Nampt: nicotinamide phosphoribosyltransferase
NF-κB: nuclear factor-κB
NMN: nicotinamide mononucleotide
NOX4: NADPH oxidase 4
NQO-1: NAD(P)H quinone dehydrogenase 1
Nrf2: nuclear factor erythroid 2–related factor 2
PAI-1: plasminogen activator inhibitor-1
p66Shc: 66 kDa Src homology 2 domain–containing protein
PGC-1α: peroxisome proliferator–activated receptor-γ coactivator 1α
PKC: protein kinase C
PPAR: peroxisome proliferator–activated receptor
PTC: proximal tubular cell
RAS: renin-angiotensin system
ROS: reactive oxygen species
Smad: small mothers against decapentaplegic homolog
SNP: single nucleotide polymorphism
SOD: superoxide dismutase
STAT3: signal transducer and activator of transcription3
T1DM: type 1 diabetes mellitus
T2DM: type 2 diabetes mellitus
TNF-α: tumor necrosis factor-α
TGF-β1: transforming growth factor-β1
USP22: ubiquitin-specific protease 22
VCAM-1: vascular cell adhesion protein-1
VEGF: vascular endothelial growth factor
